# Valley Hall Elastic Edge States in Locally Resonant Metamaterials

**DOI:** 10.3390/ma15041491

**Published:** 2022-02-17

**Authors:** Wenbo Fang, Chunyu Han, Yuyang Chen, Yijie Liu

**Affiliations:** School of Civil Engineering, Guangzhou University, Guangzhou 510006, China; fanteck@163.com (W.F.); gzhuhanchunyu@outlook.com (C.H.); yuyang199828@outlook.com (Y.C.)

**Keywords:** quantum valley Hall effect, hexagonal lattice, locally resonant metamaterial

## Abstract

This paper presents a locally resonant metamaterial periodically rearranged as a local resonator, that is hexagonal holes arranged in a thin plate replace the elastic local resonator to achieve the quantum valley Hall effect. Due to the $C_{3v}$ symmetry in the primitive hexagonal lattice, one Dirac point emerges at high symmetry points in the Brillouin zone in the sub-wavelength area. Rotating the beam element of the resonator can break the spatial inversion symmetry to lift the Dirac degeneracy and form a new bandgap. Thus, the band inversion is discovered by computing the relationship between the associated bandgap and the rotational parameter. We also confirmed this result by analyzing the vortex chirality and calculating the Chern number. We can discover two kinds of edge states in the projected band obtained by computing the supercell composed of different topological microstructures. Finally, the propagation behavior in various heterostructures at low frequencies was analyzed. It is shown that these valley Hall elastic insulators can guide elastic waves along sharp interfaces and are immune to backscattering from defects or disorder. By utilizing elastic resonators, a simple reconfigurable topological elastic metamaterial is realized in the sub-wavelength area.

## 1. Introduction

Elastic metamaterials are designed specifically to control and manipulate the propagation behavior of elastic waves. Generally, these materials can exhibit several unique characteristics, such as negative density [1,2], negative refraction [3], negative stiffness, and multistage stiffness [4], which are caused by the existence of the bandgap in the dispersion relationship. Recently, topological insulators (TIs) have offered a novel platform for wave manipulation in quantum and electronic systems [5,6,7,8,9,10]. In TIs, topologically protected edge states (TPESs) provide robust wave propagation immune to disturbances and defects. This working concept of TIs has rapidly been extended to other classical fields such as the photonics [2,3,11], acoustics [12,13,14,15], and elastic fields [16,17,18,19].

One of the first quantum topological concepts to realize elastic TIs was the quantum Hall effect (QHE). Achieving the QHE in an elastic or acoustic system requires an external field or energy input to break the time-reversal symmetry, which may be accomplished by the application of a gyroscopic inertial effect [5,6,20], a spinning fluid [7,8,9], or an external magnetic field [10,21]. However, unlike electrical systems, the complexity required to apply external energy outputs may result in QHE systems being large in size, limiting their applicability to device-level applications. Besides, researchers have shifted their focus to the elastic wave analogue of the quantum spin Hall effect (QSHE), which is able to retain the time-reversal symmetry. While these QSHE systems are passive in the conventional sense, the doubly degenerate Dirac cone generated by zone folding [22,23,24] or accidental degeneracy [24,25,26,27,28] is required for the establishment of two pseudospin states. As a consequence, their practical applications are also complicated.

The third way to achieve elastic TIs is the breaking of spatial cell symmetry to realize the quantum valley Hall effect (QVHE) [29,30,31]. The QVHE system only requires the formation of a single Dirac degeneracy in the dispersion relation. This Dirac point can be lifted by employing the passive method to form a new bandgap, which can develop the TPES for valley-dependent properties at high symmetry points in the Brillouin region [32,33,34]. Lu et al. [29] proposed a bilayer sonic crystal consisting of two-layer rotatable regular triangle scatterers to realize the layer-mixed and layer-polarized topological valley Hall phases. In the Kagome arrangement plate, Lera et al. [35] broke the inversion symmetry to research an elastic valley topological insulator. Chen et al. [36] also applied the quantum valley Hall effect to the Kagome lattice by using an asymptotic continuum model and demonstrated the immunity of the valley TPES to the scattering of various defects. In addition, topological phase transitions have been achieved by using an external force or deformation [37,38] to realize the tunable valley TPES. Nguyen et al. [38] proposed a mechanically soft phononic crystal, where the mechanical deformation may be used to control the topological behavior successfully.

Some researchers have employed the piezoelectric shunt method [39,40,41,42] to manipulate the frequency range of the valley TPES and further realize reconfigurable TIs. Zhou et al. [43] developed a tunable QVHE membrane-type metamaterial. It was found that the topological frequency range was broadened by applying an external electrical field. Riva et al. [44] utilized the negative capacitance shunt circuit to adjust the working frequency and control the propagation path of elastic waves. However, these topological effects have only been observed in high-frequency wave modes. To reduce the working frequency range, the locally resonant metamaterial can be employed to replace the traditional phononic plate in this paper. Kherraz et al. [45] realized a locally resonant effect in a single-phase phononic plate by using the distribution of circle cavities in homogeneous media. We present a locally resonant metamaterial that substitutes an elastic local resonator for hexagonal holes in a thin plate. When this hexagonal system has $C_{3v}$ symmetry, a Dirac cone occurs at the K or $K^{\prime }$ point in the Brillouin zone, which is located at the sub-wavelength frequency range after introducing the resonator. Then, altering the connecting angle of the beam element in the resonator breaks the spatial inversion symmetry to lift this single Dirac cone and generate a new bandgap, which realizes the QVHE.

This paper is organized as follows. In Section 2, the numerical models associated with these metamaterials are given, and their band structures are calculated by employing the finite element method. We also discovered the band inversion by analyzing the relationship between the design bandgap and the rotational parameter. Section 3 further confirms the topological transition by calculating the Chern number. Finally, Section 4 analyzes the wave propagation in various heterostructures with different topological microstructures. It is shown that the topologically protected edge state may sustain wave transport in the valley topology.

## 2. Numerical Model of the Locally Resonant Metamaterial

**Figure 1:**
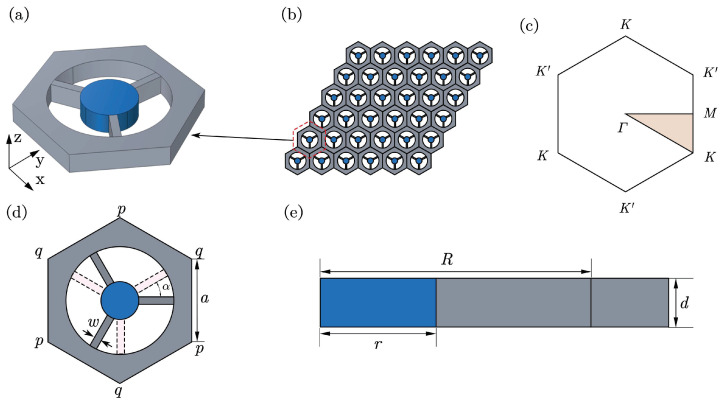
(**a**) Three-dimensional view of a primitive cell based on the hexagonal lattice. (**b**) Structural model of the locally resonant metamaterial. (**c**) Irreducible Brillouin zone. (**d**) Top view of the primitive cell. (**e**) Cutaway view of the primitive cell.

Consider a locally resonant metamaterial that periodically rearranges its local resonator by replacing a hexagonal hole in the thin plate (see Figure 1b). Figure 1c gives the first Brillouin zone corresponding to this primitive cell of Figure 1a. In Figure 1d, two adjacent corners (*p* and *q*) of the hexagon lattice represent spatially inequivalent points. When these internal beams connect at *q* points, this system has $C_{3v}$ symmetry, implying that one Dirac point occurs at high symmetry points in the Brillouin zone. We can change the rotational angle of the internal beam to lift this Dirac cone to realize the bandgap reopening and closure, where the rational parameter is defined as α. The radius (*r*) of the circular core inside the unit cell is 3.5 mm; the radius (*R*) of the round hole is 7.5 mm; the width (*w*) of the beam is 1 mm; the thickness (*d*) of the thin plate is 2 mm; the cell length (*a*) is 20 mm, as illustrated in Figure 1e. The grey and blue regions denote the rubber and lead in Figure 1, and their specific material parameters are given in Table 1.

**Figure 2:**
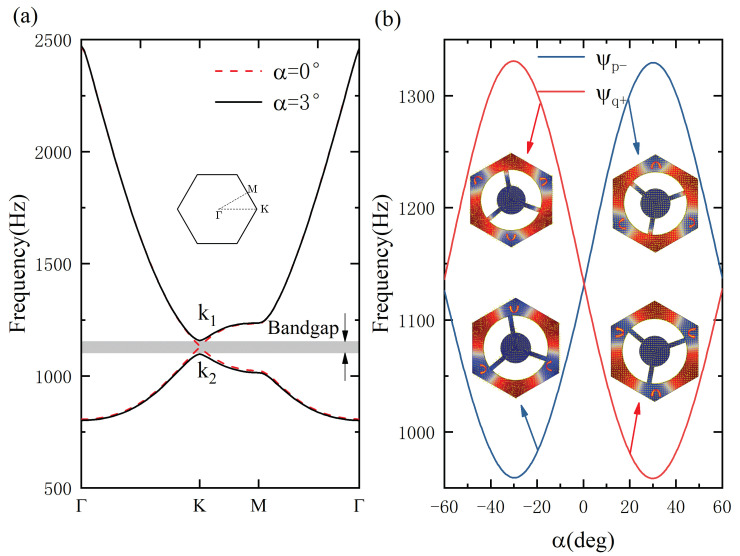
Band structure of the locally resonant metamaterial: (**a**) dispersion relationship of α=0 and $\alpha =\pm 3^{\circ }$; (**b**) relationship between the design bandgap and the rotational parameter, and vibrational modals and vortex vectors of upper and lower valleys.

The band structures in this system were calculated by using the COMSOL Multiphysics software. The red dotted lines of Figure 2a present the dispersion relationship at $\alpha =0^{\circ }$. Only the out-of-plane displacement $u_{z}$ was considered in this paper. The rational parameter α was employed to break the spatial inversion symmetry to open the Dirac point to generate a new full bandgap. Obviously, the associated bandgaps for ±α have the same frequency range and are located at the sub-wavelength zone. The result of $\alpha =3^{\circ }$ is denoted by the black line of Figure 2a. Thus, we also demonstrate the relationship between the design bandgap and the rotational parameter, as shown in Figure 2b. It is shown that the band inversion emerges in the variation of α. Figure 2b gives the vibrational modals and energy flows of the upper and lower valleys for ±α. The energy flows of the upper valley for $\alpha =-3^{\circ }$ spin clockwise around three centers at point *p*, denoted by the vortex symbol $\Psi_{p-}$. In contrast, the energy flux of the lower valley rotates around another point *q* in an anti-clockwise manner, denoted by $\Psi_{q+}$. The red and blue curves in Figure 2b denote two patterns of $\Psi_{q+}$ and $\Psi_{p-}$, respectively. The vibrational characteristics of the upper valley for −α are identical to the results of the lower valley for +α. The results of the lower valley for −α turn into those of the upper valley for +α. These discoveries further confirmed the band inversion to generate the condition to realize the QVHE.

## 3. Topological Phase

Although these band structures for ±α in Figure 2a have the same stopbands, they have different topological properties according to the principle of vortex chirality. Here, we can calculate the Chern number of the associated bandgap to confirm this discovery further. For any dispersion curve bounding the topological bandgap, the Berry curvature ($B_{n}$) is defined as in [38]:

(1) $$B_{n}\left(k\right)=\nabla k\times \langle U_{n}\left(k\right)\left|i\nabla k\right|U_{n}\left(k\right)\rangle$$

where $U_{n}\left(k\right)$ denotes the eigenmode of the $n_{\mathrm{th}}$ band calculated at the wave vector **k** and ∇ is the gradient operator.

**Figure 3:**
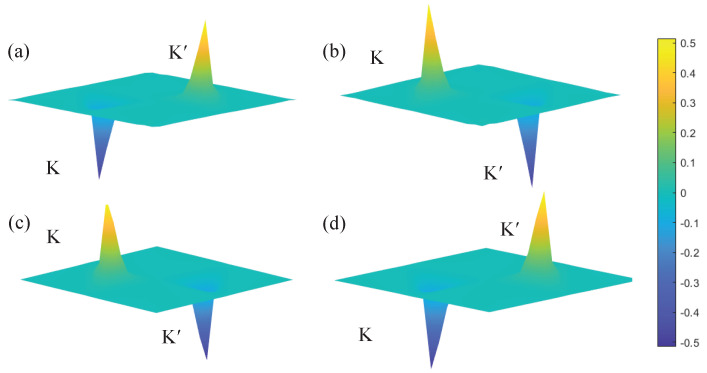
Berry curvatures: (**a**) upper valley band of $\alpha =3^{\circ }$; (**b**) lower valley band of $\alpha =3^{\circ }$; (**c**) upper valley band of $\alpha =-3^{\circ }$; (**d**) lower valley band of $\alpha =-3^{\circ }$.

The Berry curvatures for the band structures with $\alpha =\pm 3^{\circ }$ are shown in Figure 3. The $B_{n}$ values at points K and K’ have the same amplitudes and exhibit the opposite signs because this system is protected by the time inversion symmetry. The Berry curvature of the lower band for $\alpha =3^{\circ }$ around the K point is negative in Figure 3a, and the corresponding result for $\alpha =-3^{\circ }$ becomes positive in Figure 3c. This phenomenon is identical to the comparison of Figure 3b,d. These results demonstrate that two band structures for $\alpha =\pm 3^{\circ }$ exhibit different topological behaviors. Moreover, the valley Chern number $C_{v}$ can also be calculated to describe the topological characteristic of the design bandgap. The valley Chern number $C_{v-n}$ of the nth band yields:

(2) $$C_{v-n}=\frac{1}{2\pi }\int_{v}B_{n}\left(k\right)d^{2}k$$

The valley Chern numbers are computed by integrating the Berry curvature. When $\alpha =3^{\circ }$, the upper valley Chern number $C_{v-1}^{+\alpha }$ equals −0.41, which is inconsistent with the theoretical value of 0.5. This deviation originated from the strong spatial inversion symmetry breaking [46]. Thus, $C_{v-1}^{-\alpha }$,$C_{v-2}^{+\alpha }$ and $C_{v-2}^{-\alpha }$ are given as 0.41, 0.41 and −0.41, respectively. We found that the valley Chern number of the lower valley band for $\alpha =3^{\circ }$ is identical to that of the upper valley band for $\alpha =-3^{\circ }$. The result of the upper valley for $\alpha =3^{\circ }$ is the same as that of the lower valley for $\alpha =-3^{\circ }$. Meanwhile, the valley Chern number $C_{v}$ of the design bandgap is defined as:

(3) $$C_{v}=C_{v-1}-C_{v-2}$$

The valley Chern number $C_{v}$ of $\alpha =-3^{\circ }$ equals −0.82, and the calculated result of $\alpha =3^{\circ }$ becomes 0.82. Therefore, the design bandgaps of $\alpha =\pm 3^{\circ }$ have different topological properties, implying that the topological transition is developed in the variational process between $\alpha =3^{\circ }$ and $\alpha =-3^{\circ }$.

## 4. Valley Topology Edge States

**Figure 4:**
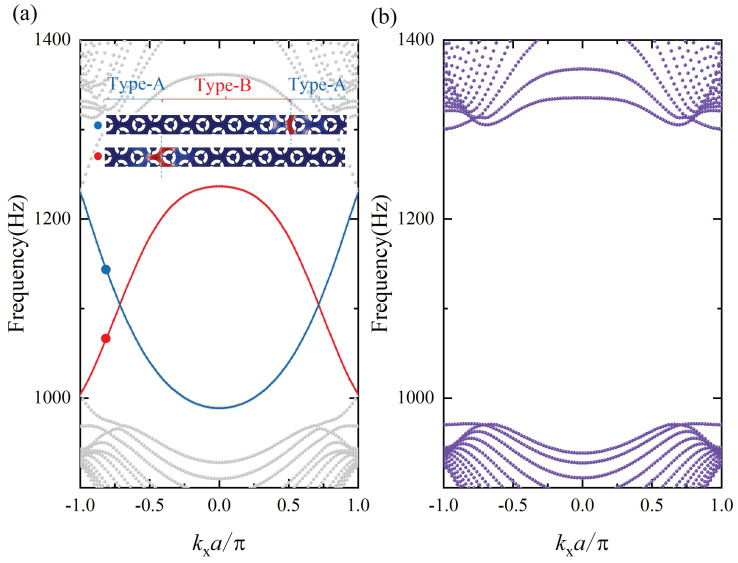
Projection band structure diagram of the supercell and its displacement field distribution: (**a**) the band structure of the ABA thin plate and the displacement field of the red and blue two points; (**b**) the band structure of all A-type thin plates.

**Figure 5:**
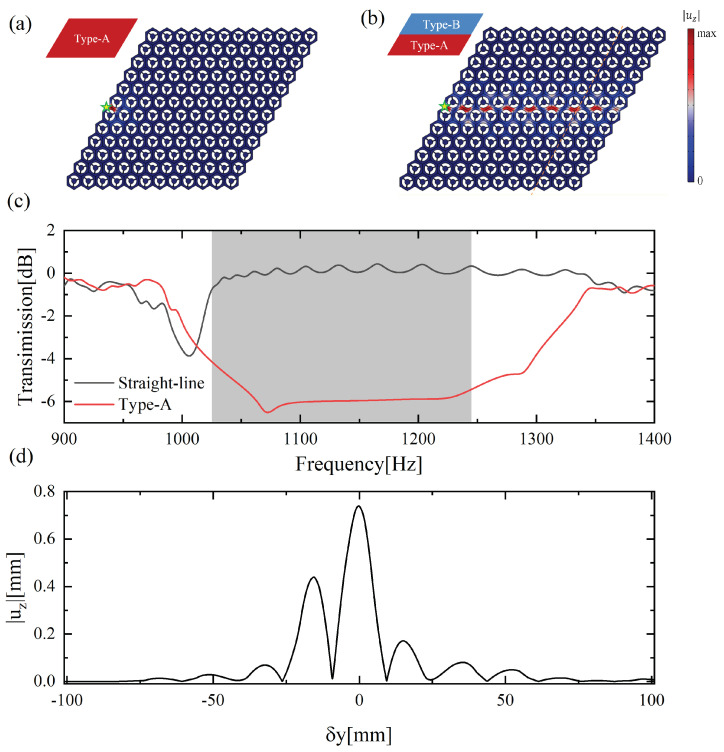
Intensity distribution of the displacement field and transmission spectrum under the out-of-plane harmonic excitation of 1133.5 Hz: (**a**) displacement field distribution of the traditional Type-A waveguide composed of entirely A-type cells, where the star denotes the excited position; (**b**) displacement field distribution of the straight-line channel topological waveguide; (**c**) transmission curves of the Type-A and straight-line waveguide; (**d**) intensity distribution of displacement |$u_{z}$| associated with the orange line range in (**b**).

According to the bulk edge correspondence principle, the edge modes merge at the domain wall between opposite topological microstructures. On the basis of the prior analysis of the valley Chen number, a supercell consisting of eight Type-A cells ($\alpha =20^{\circ }$) and eight Type-B cells ($\alpha =-20^{\circ }$) is given in Figure 4a. This superlattice has two kinds of domain walls (A-B and B-A): the A-B domain walls show that Type-A cells are directly above Type-B cells, whereas Type-B cells are above Type-A cells in the B-A domain walls. The Floquet–Bloch periodic condition is imposed along the left and right edges, whereas the perfect match layer condition applies to other edges. Figure 4a presents the projected band of the supercell calculated by the commercial finite element software COMSOL Multiphysics. The blue and red curves represent the dispersion relationships of the associated edge states located at the B-A and A-B domain walls, respectively. Dissimilarly, two modes with opposite group velocities cannot be coupled. To further verify the existence of edge states, another system consisting only of Type-A cells was considered. As shown in Figure 5b, the projected band from 1016 Hz to 1250 Hz is completely gapped to confirm the unique mechanism of the topological interface.

We carried out full-field numerical simulations under low-reflection boundary conditions to perform the robust transmission of TPEWs. Figure 5a,b denotes the Type-A and straight-line waveguides, each containing a 12 × 12 hexagonal array of cells. These waveguides were excited at the green star position in Figure 5a,b by applying an out-of-plane load at a frequency of 1133.5 Hz. It can be seen in Figure 5b that the distribution of $|u_{z}|$ is mainly concentrated at the interface. In comparison, the displacement field for the Type-A waveguide shown in Figure 5a is only localized at the excited position, indicating that the flexural elastic wave does not travel through this structure because this excited frequency is within the design bandgap. Figure 5d illustrates the distribution of the $|u_{z}|$ profile associated with the yellow cutline positions of Figure 5b. The horizontal coordinate represents the distance between the receiving position and the interface, and the vertical coordinate indicates the absolute value of the magnitude of the out-of-plane displacement. We discovered that the energy is mainly concentrated near the interface and decays rapidly to both sides of the interface. The transmission curves of these two waveguides were computed, as shown in Figure 5c. The gray region presents the design bandgap. The flexural waves can travel nicely via the B-A domain walls of the straight-line waveguide, but do not propagate in the Type-A waveguide, even if these two waveguides are excited in the same way.

**Figure 6:**
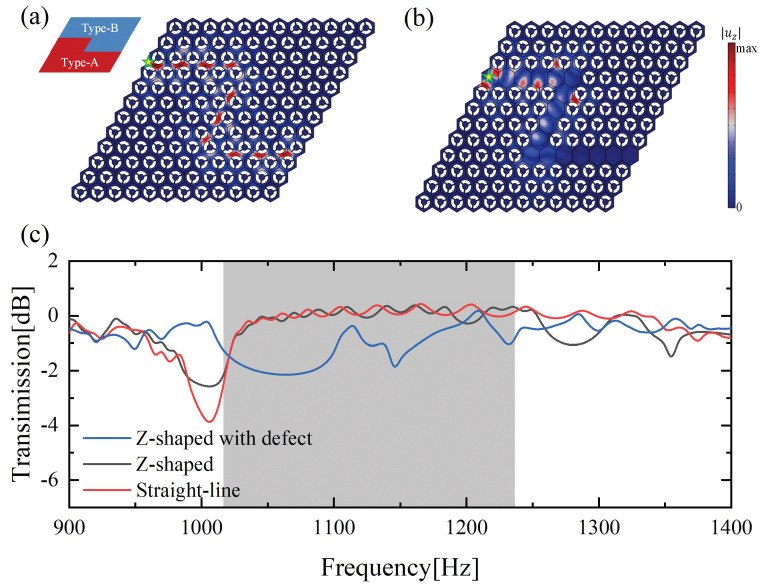
Displacement field intensity distribution and transmission curve under an out-of-plane harmonic excitation of 1133.5 Hz: (**a**) displacement field distribution of the Z-shaped waveguide; (**b**) displacement field distribution of the traditional defect waveguide with the Z-shaped channel; (**c**) transmission curve of these two structures of (**a**,**b**).

To further illustrate the anti-scattering property of the topologically protected edge state, two Z-shaped channel structures were constructed, as shown in Figure 6a,b. Their excited forms were identical to that of the straight-line waveguide. It is shown that the elastic wave propagates well along the preset interface; see Figure 6a. We discovered that the corner hardly affects the propagation of the edge state. The $|u_{z}|$ displacement profile of the traditional defect Type-A waveguide with a Z-shaped channel is shown in Figure 6b. Obviously, the flexural wave does not propagate well through the first corner and is almost blocked at this corner. In Figure 6c, the blue, black, and red curves denote the transmission of the Type-A waveguide with a Z-shaped defect, the Z-shaped waveguide, and the straight-line structure. It is shown that the transmission ratio of the former is generally lower than those of the latter two structures in the design bandgap range. Therefore, the valley topological edge state for the flexural waves has better immunity at the corner than the defect state of the traditional waveguide. The result of the Z-shaped topological structure is close to that of the straight-line structure in the bandgap frequency range. This discovery further confirms the excellent anti-scattering properties of the edge state at the corner.

**Figure 7:**
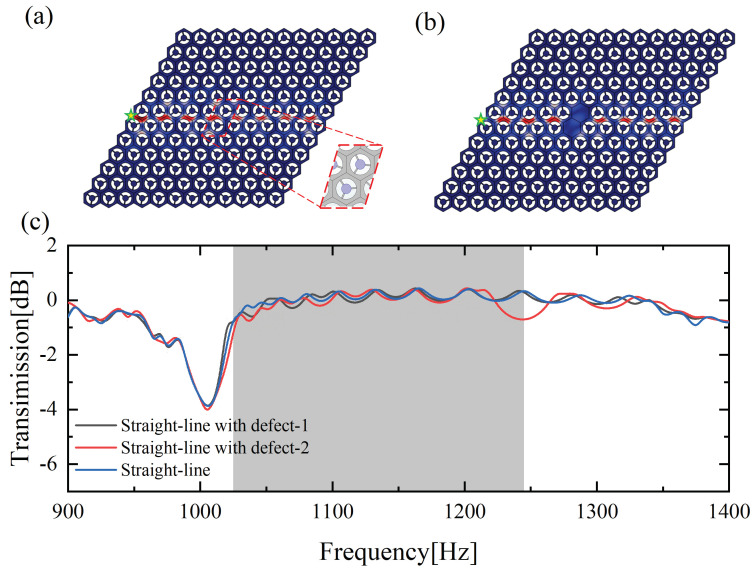
Displacement field intensity distribution and transmission curve under an out-of-plane harmonic excitation of 1133.5 Hz: (**a**) straight-line topological waveguide with disordered elements; (**b**) straight-line topological waveguide with defect elements; (**c**) transmission curve of these two structures of (**a**,**b**).

Besides, two straight-line structures for the B-A interface having disordered and defect elements were constructed in order to investigate the immune defect performance of the valley topological edge states. The cells within the disorder range are only required to be retained in the primitive $C_{3v}$ system, as shown in Figure 7a. Figure 7b shows the defect elements, where the hexagonal plate elements were employed to replace Type-A or Type-B elements in the dashed box. By applying an out-of-plane simple harmonic excitation with a frequency of 1133.5 Hz at the green star position, the $|u_{z}|$ displacements remain concentrated on the interface, and the elastic wave propagation along the interface is not attenuated by the disorder and defect element. Meanwhile, Figure 7c presents the transmission curves of these two structures and the intact straight-line waveguide. The results of these three topological structures tend to be consistent in the bandgap frequency range, implying that the TPEWs have robustness against the presence of disorder and defects.

## 5. Conclusions

In this paper, we proposed a locally resonant metamaterial that periodically rearranges its local resonator by replacing the hexagonal hole in a thin plate to achieve the quantum valley Hall effect. The primitive cell of this locally resonant metamaterial exhibits $C_{3v}$ symmetry, meaning that one Dirac cone exists at point K or point K’ in the hexagonal Brillouin zone. Applying the rational parameter breaks the spatial inversion symmetry to lift the Dirac degeneracy and generate a new bandgap. Thus, the band inversion is developed during the variational process of the rational parameter. This phenomenon was confirmed further by using the vortex chirality. We calculated the Berry curvatures and valley Chern numbers associated with the corresponding band structures to demonstrate the topological transition, implying the existence of the TPES in this system. A full-field numerical simulation was employed to validate the propagation behavior of the TPEWs along two channels: a straight-line and a Z-shaped channel. In each channel configuration, the flexural wave traveled well along the topological interface with a limited propagation into the bulk. Meanwhile, compared with traditional waveguides, the elastic valley Hall insulator was immune to backscattering wave propagation against sharp corners, disordered elements, and defect elements. This topological transport phenomenon is expected to be applied to new acoustic devices such as directional waveguides.

## Figures and Tables

**Table 1:** The corresponding material parameters of the thin plate metamaterial.

Material	Density (kg/m^3^)	Young’s Modulus (Pa)	Poisson’s Ratio
Lead	11,300	17 × 10${}^{9}$	0.33
Rubber	115	8 × 10${}^{6}$	0.33

## Data Availability

The data that support the findings of this study are available from the corresponding author upon reasonable request.

## References

[1] Li J., Chan C.T. (2004). Double-negative acoustic metamaterial. Phys. Rev. E.

[2] Liu X.N., Hu G.K., Huang G.L., Sun C.T. (2011). An elastic metamaterial with simultaneously negative mass density and bulk modulus. Appl. Phys. Lett..

[3] Cummer S.A., Schurig D. (2007). One path to acoustic cloaking. New J. Phys..

[4] Wen G., Chen G., Long K., Wang X., Liu J., Xie Y.M. (2021). Stacked-origami mechanical metamaterial with tailored multistage stiffness. Mater. Des..

[5] Nash L.M., Kleckner D., Read A., Vitelli V., Turner A.M., Irvine W.T. (2015). Topological mechanics of gyroscopic metamaterials. Proc. Natl. Acad. Sci. USA.

[6] Fleury R., Sounas D.L., Sieck C.F., Haberman M.R., Alù A. (2014). Sound isolation and giant linear nonreciprocity in a compact acoustic circulator. Science.

[7] Ding Y., Peng Y., Zhu Y., Fan X., Yang J., Liang B., Zhu X., Wan X., Cheng J. (2019). Experimental demonstration of acoustic Chern insulators. Phys. Rev. Lett..

[8] Gao P., Zhang Z., Christensen J. (2020). Sonic valley-Chern insulators. Phys. Rev. B.

[9] Prodan E., Prodan C. (2009). Topological phonon modes and their role in dynamic instability of microtubules. Phys. Rev. Lett..

[10] Wang Z., Chong Y., Joannopoulos J.D., Soljačić M. (2008). Reflection-free one-way edge modes in a gyromagnetic photonic crystal. Phys. Rev. Lett..

[11] Ho K., Chan C.T., Soukoulis C.M. (1990). Existence of a photonic gap in periodic dielectric structures. Phys. Rev. Lett..

[12] Xu K., Jiang C. (2020). Broadening bandgap of thermocrystal by tailoring air hole. Appl. Phys. Express.

[13] Rustamov F., Darvishov N., Mamedov M., Bobrova E., Qafarova H. (2011). Porous silicon bandgap broadening at natural oxidation. J. Lumin..

[14] Xu J., Yan R., Tang J. (2018). Broadening bandgap width of piezoelectric metamaterial by introducing cavity. Appl. Sci..

[15] Xu K., Jiang C. (2020). Expanding the bandgap of thermal phonons by using supercrystals. Results Phys..

[16] Xiao Y., Wen J., Wang G., Wen X. (2013). Theoretical and experimental study of locally resonant and Bragg band gaps in flexural beams carrying periodic arrays of beam-like resonators. J. Vib. Acoust..

[17] Xiao Y., Wen J., Wen X. (2012). Flexural wave bandgaps in locally resonant thin plates with periodically attached spring–mass resonators. J. Phys. D Appl. Phys..

[18] Wang H., Liu D., Fang W., Lin S., Liu Y., Liang Y. (2020). Tunable topological interface states in one-dimensional extended granular crystals. Int. J. Mech. Sci..

[19] Liu Y., Jin L., Wang H., Liu D., Liang Y. (2021). Topological interface states in translational metamaterials for sub-wavelength in-plane waves. Int. J. Mech. Sci..

[20] Mitchell N.P., Nash L.M., Irvine W.T. (2018). Tunable band topology in gyroscopic lattices. Phys. Rev. B.

[21] Hafezi M., Demler E.A., Lukin M.D., Taylor J.M. (2011). Robust optical delay lines with topological protection. Nat. Phys..

[22] Deng Y., Ge H., Tian Y., Lu M., Jing Y. (2017). Observation of zone folding induced acoustic topological insulators and the role of spin-mixing defects. Phys. Rev. B.

[23] Zhang Z., Wei Q., Cheng Y., Zhang T., Wu D., Liu X. (2017). Topological creation of acoustic pseudospin multipoles in a flow-free symmetry-broken metamaterial lattice. Phys. Rev. Lett..

[24] Li Y., Wu Y., Mei J. (2014). Double Dirac cones in phononic crystals. Appl. Phys. Lett..

[25] Chen Z.G., Ni X., Wu Y., He C., Sun X.C., Zheng L.Y., Lu M.H., Chen Y.F. (2014). Accidental degeneracy of double Dirac cones in a phononic crystal. Sci. Rep..

[26] Xia J.P., Jia D., Sun H.X., Yuan S.Q., Ge Y., Si Q.R., Liu X.J. (2018). Programmable coding acoustic topological insulator. Adv. Mater..

[27] Chen H., Nassar H., Norris A.N., Hu G., Huang G. (2018). Elastic quantum spin Hall effect in kagome lattices. Phys. Rev. B.

[28] Darabi A., Leamy M.J. (2019). Tunable nonlinear topological insulator for acoustic waves. Phys. Rev. Appl..

[29] Lu J., Qiu C., Ke M., Liu Z. (2016). Valley vortex states in sonic crystals. Phys. Rev. Lett..

[30] Zhang Q., Chen Y., Zhang K., Hu G. (2020). Dirac degeneracy and elastic topological valley modes induced by local resonant states. Phys. Rev. B.

[31] Zhang Z., Gu Y., Long H., Cheng Y., Liu X., Christensen J. (2019). Subwavelength acoustic valley-Hall topological insulators using soda cans honeycomb lattices. Research.

[32] Zhang Z., Tian Y., Cheng Y., Wei Q., Liu X., Christensen J. (2018). Topological acoustic delay line. Phys. Rev. Appl..

[33] Tian Z., Shen C., Li J., Reit E., Bachman H., Socolar J.E., Cummer S.A., Huang T.J. (2020). Dispersion tuning and route reconfiguration of acoustic waves in valley topological phononic crystals. Nat. Commun..

[34] Ma J., Sun K., Gonella S. (2019). Valley hall in-plane edge states as building blocks for elastodynamic logic circuits. Phys. Rev. Appl..

[35] Lera N., Torrent D., San-Jose P., Christensen J., Alvarez J.V. (2019). Valley Hall phases in kagome lattices. Phys. Rev. B.

[36] Chen H., Nassar H., Huang G. (2018). A study of topological effects in 1D and 2D mechanical lattices. J. Mech. Phys. Solids.

[37] Liu T.W., Semperlotti F. (2018). Tunable acoustic valley—Hall edge states in reconfigurable phononic elastic waveguides. Phys. Rev. Appl..

[38] Nguyen B., Zhuang X., Park H., Rabczuk T. (2019). Tunable topological bandgaps and frequencies in a pre-stressed soft phononic crystal. J. Appl. Phys..

[39] Darabi A., Collet M., Leamy M.J. (2020). Experimental realization of a reconfigurable electroacoustic topological insulator. Proc. Natl. Acad. Sci. USA.

[40] Bacigalupo A., De Bellis M.L., Misseroni D. (2020). Design of tunable acoustic metamaterials with periodic piezoelectric microstructure. Extrem. Mech. Lett..

[41] Liu Y., Wang H., Fang W., Han Q., Liu D., Liang Y. (2021). Tunable control of subwavelength topological interface modes in locally resonance piezoelectric metamaterials. Compos. Struct..

[42] Liu Y., Fang W., Liang Y., Liu D., Han Q. (2021). Tuning of subwavelength topological interface states in locally resonant metastructures with shunted piezoelectric patches. J. Appl. Phys..

[43] Zhou W., Su Y., Chen W., Lim C. (2020). Voltage-controlled quantum valley Hall effect in dielectric membrane-type acoustic metamaterials. Int. J. Mech. Sci..

[44] Riva E., Quadrelli D., Cazzulani G., Braghin F. (2018). Tunable in-plane topologically protected edge waves in continuum Kagome lattices. J. Appl. Phys..

[45] Kherraz N., Radzieński M., Mazzotti M., Kudela P., Bosia F., Gliozzi A., Misseroni D., Pugno N., Ostachowicz W., Miniaci M. (2021). Experimental full wavefield reconstruction and band diagram analysis in a single-phase phononic plate with internal resonators. J. Sound Vib..

[46] Zhang Q., Chen Y., Zhang K., Hu G. (2019). Programmable elastic valley Hall insulator with tunable interface propagation routes. Extrem. Mech. Lett..

